# Advances in First-Line Treatment of Classical Hodgkin Lymphoma in the Era of Novel Agents

**DOI:** 10.3390/jcm15135075

**Published:** 2026-06-29

**Authors:** Ahmed Salleh Barefah

**Affiliations:** 1 Hematology Department, Faculty of Medicine, King Abdulaziz University Hospital, King Abdulaziz University, Jeddah 21589, Saudi Arabia; asbarefah@kau.edu.sa; 2 Hematology Research Unit, King Fahad Medical Research Center, King Abdulaziz University, Jeddah 21859, Saudi Arabia

**Keywords:** classical Hodgkin lymphoma, brentuximab vedotin, nivolumab, pembrolizumab, frontline therapy, PET-adapted treatment

## Abstract

Classical Hodgkin lymphoma (cHL) remains one of the most curable hematologic malignancies, with long-term survival exceeding 80–90% in most contemporary series. However, a subset of patients experience primary refractory disease, relapse, treatment-related toxicity, or late complications associated with conventional chemotherapy and radiotherapy. Over the last decade, major advances in frontline treatment have transformed the therapeutic landscape of cHL through the incorporation of targeted therapy and immune checkpoint inhibition into first-line regimens. Brentuximab vedotin (BV), an anti-CD30 antibody–drug conjugate, and programmed death-1 (PD-1) inhibitors such as nivolumab and pembrolizumab have significantly improved outcomes in advanced-stage disease and are increasingly being explored in limited-stage settings. The ECHELON-1 trial established BV-AVD as superior to ABVD in advanced-stage disease with improved progression-free survival and overall survival, while the SWOG S1826 study demonstrated superior progression-free survival and reduced toxicity with nivolumab-AVD compared with BV-AVD. Based on the SWOG S1826 data, nivolumab-AVD is emerging as the preferred frontline standard for most fit patients with advanced-stage cHL, though this remains an area of evolving practice. PET-adapted strategies remain a critical backbone of treatment optimization even in the era of novel agents, enabling individualized de-escalation or escalation based on early metabolic response. These advances have shifted frontline treatment paradigms toward chemotherapy de-escalation, PET-adapted strategies, and immune-based treatment approaches. In parallel, the role of radiotherapy continues to evolve with efforts aimed at minimizing long-term toxicity without compromising cure rates, particularly in PET-negative patients with limited-stage disease. This review summarizes the biological rationale, pivotal clinical trials, evolving treatment strategies, current guideline recommendations, and future directions in first-line treatment of cHL, with emphasis on evidence-based incorporation of novel agents and practical implications for modern clinical practice.

## 1. Introduction

Classical Hodgkin lymphoma (cHL) is a highly curable B-cell malignancy characterized by the presence of Reed–Sternberg cells within a rich inflammatory tumor microenvironment. Contemporary treatment strategies have resulted in cure rates exceeding 90% in early-stage disease and approximately 80–90% in advanced-stage disease. Nevertheless, treatment-related toxicities including pulmonary toxicity, infertility, secondary malignancies, cardiovascular disease, and neuropathy remain major concerns, particularly in younger patients with long anticipated survival [1,2,3].

For decades, ABVD (doxorubicin, bleomycin, vinblastine, dacarbazine) represented the standard frontline regimen for most patients with cHL owing to its favorable balance between efficacy and toxicity compared with more intensive regimens such as escalated BEACOPP. While escalated BEACOPP improved disease control in selected high-risk patients, its use has been limited by substantial hematologic toxicity, infertility risk, infections, and secondary leukemia [4].

The introduction of positron emission tomography (PET)-adapted treatment represented a major milestone in risk-adapted therapy, enabling treatment escalation or de-escalation based on early metabolic response [5]. More recently, targeted therapy and immunotherapy have revolutionized the treatment landscape of cHL. Brentuximab vedotin (BV), an anti-CD30 antibody–drug conjugate, and PD-1 inhibitors, including nivolumab and pembrolizumab, demonstrated remarkable activity in relapsed/refractory disease and subsequently moved into frontline settings [6,7].

The unique biology of cHL makes it especially susceptible to both anti-CD30 targeted therapy and PD-1 immune checkpoint inhibition. Reed–Sternberg cells universally express CD30, enabling highly selective antibody–drug conjugate delivery, while near-universal 9p24.1 amplification drives PD-L1/PD-L2 overexpression and profound susceptibility to checkpoint blockade. These features distinguish cHL from most other lymphoid malignancies and explain the exceptional single-agent and combination activity observed with brentuximab vedotin and PD-1 inhibitors across disease stages. Furthermore, the predominantly adolescent and young adult (AYA) population affected by cHL creates a compelling unmet need for regimens that achieve a durable cure while minimizing long-term toxicity, particularly infertility, cardiovascular disease, secondary malignancies, and neuropathy in patients expected to survive for decades after treatment [8].

The ECHELON-1 study established BV-AVD as a frontline standard in advanced-stage cHL with improvement in progression-free survival and overall survival compared with ABVD [7,9]. Subsequently, the SWOG S1826 trial demonstrated that nivolumab AVD achieved superior progression-free survival with a more favorable toxicity profile than BV AVD, positioning PD-1-based therapy as a potentially new frontline standard [10].

These advances have fundamentally changed the treatment paradigm of cHL and raised important questions regarding optimal sequencing, radiotherapy integration, toxicity management, survivorship, and biomarker-driven treatment selection. This review summarizes current evidence regarding frontline treatment advances in cHL with emphasis on novel-agent integration and evolving therapeutic strategies. The pediatric population (children younger than 12 years) represents a distinct patient group managed according to specific pediatric oncology protocols and dedicated treatment frameworks, and is therefore outside the scope of this review.

## 2. Biological Rationale for Novel Agents in cHL

The biology of cHL uniquely supports susceptibility to both CD30-targeted therapy and immune checkpoint blockade. Reed–Sternberg cells universally express CD30, making it an attractive therapeutic target. Brentuximab vedotin combines an anti-CD30 monoclonal antibody with the antimicrotubule agent monomethyl auristatin E, allowing selective intracellular delivery of cytotoxic therapy [6].

In addition, cHL demonstrates marked dependence on immune evasion pathways. Genetic amplification of chromosome 9p24.1, present in virtually all cHL cases, leads to copy number-dependent overexpression of both PD-L1 and PD-L2 on Reed–Sternberg cells. This amplification not only increases ligand density on tumor cells but also upregulates the JAK2-STAT signaling pathway, further potentiating PD-L1/PD-L2 expression. The resulting exhaustion of tumor-infiltrating T-cells and suppression of immune surveillance explain the profound sensitivity of cHL to PD-1 blockade [8]. Beyond their mechanistic role, 9p24.1 amplification and PD-L1 expression have been explored as potential predictive biomarkers for patient selection in PD-1 inhibitor-based frontline therapy. Higher degrees of 9p24.1 amplification correlate with greater PD-L1 overexpression and have been associated with superior response to checkpoint inhibitors in relapsed/refractory disease. Their prospective validation as predictive biomarkers in frontline immunotherapy-based regimens represents an active area of investigation that may enable future biomarker-driven patient stratification [2,8].

The integration of these agents into frontline therapy has allowed improvement in disease control while potentially reducing reliance on highly toxic chemotherapy regimens and radiotherapy.

Also, the role of Epstein–Barr virus (EBV) status as a potential predictive biomarker for immunotherapy response in cHL is an emerging area of investigation. EBV-positive cHL is characterized by distinct tumor microenvironment features, including greater infiltration of EBV-specific cytotoxic T lymphocytes and differential expression of immune checkpoints. Emerging data suggest that EBV positivity in cHL may be associated with differential outcomes with PD-1 inhibitor-based therapy; however, findings across studies have been inconsistent and the precise clinical significance of EBV status in the context of frontline immunotherapy remains to be prospectively validated. Consideration of EBV status as an exploratory predictive biomarker in ongoing and future frontline PD-1 inhibitor trials is warranted [11].

## 3. Evolution of Frontline Therapy in Classical Hodgkin Lymphoma ABVD Era

ABVD became the standard frontline regimen following randomized studies demonstrating superior efficacy and lower toxicity compared with MOPP-containing regimens [12]. Long-term outcomes with ABVD showed durable remission rates with relatively acceptable toxicity profiles. However, bleomycin pulmonary toxicity remained a significant limitation, particularly among older patients and those receiving growth factor support [13]. Furthermore, approximately 20–30% of advanced-stage patients experienced relapse or refractory disease following ABVD [2,14].

## 4. Escalated BEACOPP

Escalated BEACOPP improved progression-free survival compared with ABVD in advanced-stage disease, particularly among high-risk patients [15]. Longterm follow-up from the German Hodgkin Study Group confirmed superior disease control but at the expense of increased infertility, infections, myelodysplasia, and secondary acute leukemia [4].

In contemporary practice, the role of escalated BEACOPP in advanced-stage cHL has been substantially redefined by the emergence of less toxic yet highly effective novel-agent-based regimens. Escalated BEACOPP retains disease control superiority over ABVD in high-risk settings, but its severe acute and long-term toxicity burden, including febrile neutropenia, infertility, myelodysplasia, and secondary leukemia, continues to restrict its use to a carefully selected subset of fit younger patients, particularly those with IPS ≥ 4 [15]. The most recent ESMO Clinical Practice Guideline (2025) now positions eBEACOPP as a fallback option applicable only when both BV and PD-1 inhibitors are unavailable and assigning it a lower evidence rating [IV, B] compared with the preferred regimens of nivolumab-AVD and BrECADD [16]. The NCCN Guidelines (Version 1.2026) have similarly removed escalated BEACOPP as a recommended option, with BrECADD effectively replacing it as the intensive treatment choice for fit patients with advanced-stage disease [17]. This paradigm shift reflects the results of the GHSG HD21 trial, in which BrECADD demonstrated superior tolerability with significantly lower treatment-related morbidity alongside non-inferior and numerically superior progression-free survival (4-year PFS 94.3% vs. 90.9%) compared with escalated BEACOPP, establishing BrECADD as the preferred intensive frontline regimen for fit younger patients with very high-risk advanced-stage cHL requiring an intensive treatment approach [18].

## 5. PET-Adapted Therapy

Interim PET imaging became central to frontline treatment decisions after studies demonstrated the strong prognostic significance of early metabolic response [19]. The RATHL trial demonstrated that bleomycin could safely be omitted after negative interim PET following two cycles of ABVD, significantly reducing pulmonary toxicity without compromising outcomes [5]. PET-adapted escalation strategies similarly improved outcomes in PET-positive patients through treatment intensification.

Historically, PET-adapted approaches were developed in the chemotherapy era, particularly with ABVD and escalated BEACOPP, where interim PET findings allowed intensification of treatment in high-risk patients and reduction in treatment-related toxicity in early responders. In the modern era of novel-agent-based frontline therapy, including brentuximab vedotin plus AVD (BV-AVD), nivolumab plus AVD (Nivo-AVD), and BrECADD, the role of interim PET-CT is evolving. Data from ECHELON-1 demonstrated the reduced necessity for PET-directed escalation with BV-AVD due to improved disease control compared with ABVD, while studies such as S1826 have shown remarkably high progression-free survival rates with Nivo-AVD irrespective of interim PET status, raising questions about the predictive value of PET in the context of immune checkpoint blockade [9,10]. Furthermore, immunotherapy introduces unique challenges, including inflammatory responses and pseudo-progression, which may complicate PET interpretation and limit its specificity [20]. Consequently, current research is shifting from using interim PET solely as a trigger for treatment escalation toward employing it as a tool for treatment de-escalation, therapy shortening, and minimizing long-term toxicity in patients achieving rapid metabolic responses. Future strategies are likely to integrate PET-CT with emerging biomarkers such as circulating tumor DNA (ctDNA), molecular response assessment, and artificial intelligence-based imaging analysis to create more refined risk-adapted approaches, ultimately enabling personalized treatment while maintaining the excellent outcomes achieved with modern regimens [21,22,23,24].

## 6. Brentuximab Vedotin in Frontline Therapy

### ECHELON-1 Trial

The phase III ECHELON-1 trial represented a major advance in frontline treatment. This study randomized patients with stage III–IV cHL to BV-AVD versus standard ABVD. BV-AVD demonstrated improved modified progression-free survival compared with ABVD [9]. Updated long-term analysis further demonstrated overall survival benefit favoring BV-AVD, with 6-year overall survival rates of 93.9% versus 89.4% for ABVD [7].

Peripheral neuropathy and neutropenia were more common with BV-AVD, and G-CSF prophylaxis is recommended to mitigate febrile neutropenia risk. Although many neuropathy cases improved or resolved with time and dose modification, cumulative neurotoxicity is particularly relevant when BV may be reused in salvage settings [9]. The ECHELON-1 trial established BV-AVD as a frontline standard for advanced-stage cHL and marked the first significant survival improvement over ABVD in decades [7]. However, with the emergence of robust data from the SWOG S1826 trial demonstrating the superiority of nivolumab-AVD and the favorable outcomes reported with BrECADD in the GHSG HD21 trial, both the ESMO and NCCN guidelines have repositioned BV-AVD as a secondary preferred regimen. BV-AVD now serves as an excellent alternative when nivolumab-AVD is unavailable or contraindicated, or when an intensified BrECADD-based approach is not appropriate because of patient characteristics or treatment-related considerations. Despite this shift, BV-AVD remains a well-established frontline option supported by mature long-term efficacy and overall survival data and continues to have an important role in the contemporary management of advanced classical Hodgkin lymphoma [16,17].

## 7. GHSG HD21 Trial (BrECADD Study)

More recently, the German Hodgkin Study Group HD21 trial evaluated the novel BrECADD regimen (brentuximab vedotin, etoposide, cyclophosphamide, doxorubicin, dacarbazine, dexamethasone) compared with escalated BEACOPP in patients with advanced-stage cHL. The study demonstrated that BrECADD achieved superior tolerability with reduced treatment-related morbidity and fewer serious adverse events while maintaining excellent disease control. Importantly, BrECADD was associated with lower hematologic toxicity, reduced infertility risk markers, and fewer treatment discontinuations compared with escalated BEACOPP [18]. BrECADD showed non-inferior efficacy with excellent progression-free survival outcomes and significantly lower treatment-related toxicity, supporting its role as a potential new intensive-treatment standard for younger fit patients with advanced-stage disease [18].

Furthermore, the PET-adapted design of HD21 enabled treatment de-escalation, whereby patients who achieved a negative PET scan after two cycles of BrECADD received only two additional cycles (four cycles in total), while only PET-positive patients continued to receive six total cycles. This strategy substantially reduced cumulative chemotherapy exposure and treatment duration without compromising efficacy [18]. The PET-adapted BrECADD strategy represents an important evolution toward response-adapted therapy, allowing the majority of patients to receive a shorter treatment course with less exposure to alkylating agents and anthracyclines while preserving outstanding clinical outcomes. This reduction in cumulative chemotherapy may translate into lower risks of long-term toxicities, including infertility, secondary malignancies, and cardiopulmonary complications, making BrECADD an attractive option for younger patients expected to achieve long-term survivorship [18].

BrECADD is recognized in both the ESMO and NCCN guidelines as a preferred frontline option for younger, fit patients with advanced-stage classical Hodgkin lymphoma, particularly as an effective PET-adapted intensified regimen that offers excellent disease control while reducing treatment-related toxicity compared with escalated BEACOPP [16,17].

## 8. PD-1 Inhibitors in Frontline Therapy

### Nivolumab-AVD and SWOG S1826

The SWOG S1826 trial represented a landmark study in frontline classical Hodgkin lymphoma (cHL) treatment by comparing nivolumab-AVD with BV-AVD in adolescents and adults with advanced-stage disease (Herrera et al., 2024) [10]. This large multicenter phase III trial enrolled more than 900 patients with stage III–IV cHL and demonstrated that nivolumab-AVD significantly improved progression-free survival compared with BV-AVD. The 2-year progression-free survival was 92% in the nivolumab-AVD arm versus 83% in the BV-AVD arm (hazard ratio 0.45), representing one of the most favorable frontline outcomes ever reported in advanced-stage cHL [10].

Importantly, the benefit of nivolumab-AVD was consistent across multiple predefined subgroups, including older patients, adolescents, and patients with high-risk International Prognostic Score (IPS) features. In addition, the regimen demonstrated lower rates of peripheral neuropathy, febrile neutropenia, and treatment discontinuation compared with BV-AVD [10].

Another important observation from the study was the reduced reliance on consolidative radiotherapy, with only a very small proportion of patients requiring radiation at the end of therapy, supporting the concept that highly active immunochemotherapy combinations may reduce long-term radiation-associated toxicity [10].

Furthermore, nivolumab-AVD demonstrated favorable tolerability despite the omission of routine growth factor support in many patients, and immune-related adverse events were generally manageable and infrequently led to treatment discontinuation.

These findings established PD-1 blockade as a highly effective frontline strategy and suggested that immune-based approaches may outperform antibody–drug conjugate-based therapy in advanced-stage cHL. The SWOG S1826 study is now considered practice-changing and has positioned nivolumab-AVD among the preferred frontline regimens in contemporary NCCN and ESMO recommendations for advanced-stage cHL [16,17].

## 9. Pembrolizumab-Based Frontline Strategies

Pembrolizumab has similarly shown promising frontline activity. Early-phase studies evaluating pembrolizumab sequentially or concurrently with AVD demonstrated high complete response rates and durable remissions [25]. However, it is important to note that pembrolizumab-based frontline regimens for advanced-stage cHL remain supported primarily by phase II data and are not yet validated by phase III randomized evidence. Accordingly, pembrolizumab-based frontline approaches should currently be regarded as investigational in the advanced-stage setting, distinct from nivolumab-AVD, which has phase III support from SWOG S1826.

The favorable toxicity profile and impressive efficacy of PD-1 blockade have generated interest in chemotherapy minimization and potentially chemotherapy-free approaches in selected patients.

## 10. SGN35-027 Trial: BV Plus Nivolumab Combination

The SGN35-027 trial is a phase II study evaluating the combination of brentuximab vedotin plus nivolumab with doxorubicin and dacarbazine (AN + AD; vinblastine omitted) as frontline therapy in previously untreated advanced-stage cHL. This novel approach simultaneously targets CD30 via antibody–drug conjugate delivery and disrupts the PD-1/PD-L1 immune evasion axis, potentially achieving synergistic antitumor activity through complementary mechanisms. Preliminary results from the advanced-stage cohort demonstrated an overall response rate of approximately 93–95% with a complete response rate of approximately 88–89% and a 2-year progression-free survival of approximately 88%, supporting the feasibility and activity of this chemotherapy-minimized doubly targeted strategy. An early-stage cohort has also been evaluated with similarly encouraging response rates (~95% ORR, −92% CR). While these data remain early phase and require confirmation in randomized phase III studies, the SGN35-027 trial provides important proof-of-concept data for combining BV and checkpoint inhibition in the frontline setting and may have meaningful implications for future chemotherapy-free or chemotherapy-minimized treatment strategies in cHL [26].

## 11. Novel Agents in Limited-Stage Disease

The incorporation of novel agents into limited-stage disease remains an active area of investigation. Limited-stage cHL is conventionally divided into favorable and unfavorable subgroups, which differ in their risk factor burden, treatment intensity, and likelihood of radiotherapy omission being safely explored. Favorable early-stage cHL (generally defined by the absence of risk factors such as large mediastinal mass, extranodal disease, elevated ESR, or ≥3 involved sites) achieves excellent outcomes with abbreviated ABVD and involved-site radiotherapy. Unfavorable early-stage cHL carries a higher relapse risk and typically requires more intensive treatment, making it the primary focus of novel-agent integration in the limited-stage setting.

Currently, for limited-stage classical Hodgkin lymphoma, the 2025 ESMO guideline recommends 2 cycles of ABVD followed by 20 Gy ISRT for favorable disease, while unfavorable disease is treated with either 4 cycles of ABVD followed by 30 Gy ISRT or, in fit patients ≤ 60 years, 2 cycles of escalated BEACOPP followed by 2 cycles of ABVD, with PET-adapted omission or use of 30 Gy ISRT. The 2026 NCCN guideline remains broadly aligned, using combined-modality therapy with ABVD-based chemotherapy plus ISRT, typically 2 cycles ABVD + ISRT for favorable disease and 4 cycles ABVD + ISRT or selected PET-adapted systemic approaches for unfavorable disease [16,17].

Efforts primarily focus on reducing radiotherapy exposure, improving early PET negativity and minimizing long-term toxicity. Several studies evaluating BV or PD-1 inhibitors combined with abbreviated chemotherapy have demonstrated excellent response rates and encouraging progression-free survival [27].

The phase II BREACH trial evaluated BV-AVD versus ABVD in patients with early-stage unfavorable classical Hodgkin lymphoma (cHL), followed by involved node radiotherapy. BV-AVD demonstrated improved PET negativity after two cycles compared with ABVD (82.3% vs. 75.4%) and showed excellent 2-year progression-free survival of 97.3%, supporting the activity of BV-based therapy in limited-stage disease [28].

The phase II German Hodgkin Study Group NIVAHL trial evaluated nivolumab combined with AVD using either sequential or concomitant schedules in patients with early-stage unfavorable cHL followed by involved-site radiotherapy. The study demonstrated very high complete remission rates, high PET-negativity rates, and excellent survival outcomes, supporting PD-1 blockade as a promising frontline strategy in limited-stage disease [29].

Additional early-phase pembrolizumab-based frontline studies have explored sequential pembrolizumab followed by AVD or concurrent pembrolizumab-containing approaches, demonstrating encouraging metabolic response rates and durable short-term disease control. However, these studies remain less mature and continue to be considered investigational in limited-stage disease [25].

Together, these studies suggest that BV- and PD-1-based frontline approaches may improve early metabolic response and potentially allow reduction or omission of radiotherapy in carefully selected PET-negative patients. Nevertheless, longer follow-up and randomized phase III studies are needed before radiotherapy omission can be considered standard practice.

A summary of the pivotal frontline trials evaluating brentuximab vedotin- and PD-1 inhibitor-based strategies in advanced-stage and limited-stage classical Hodgkin lymphoma is presented in Table 1 and Table 2, respectively.

## 12. Role of Radiation Therapy in Early-Stage cHL: RT Alone vs. Sequential Combined-Modality Therapy

In early-stage cHL, sequential combined-modality therapy (CMT) chemotherapy followed by involved-site radiotherapy (ISRT) remains the standard of care for most patients. For favorable limited-stage disease (stages I–II without risk factors), the GHSG HD10 trial established two cycles of ABVD followed by 20 Gy ISRT as standard, with outcomes equivalent to more intensive approaches and reduced toxicity. For unfavorable early-stage disease, four cycles of ABVD followed by 30 Gy ISRT is standard. For bulky mediastinal disease (≥10 cm or mediastinal mass ratio ≥ 0.33), consolidative ISRT at 30–36 Gy is generally recommended given the high risk of local relapse after chemotherapy alone [14].

Radiation therapy alone remains a less favored option only in highly selected patients with favorable stage IA non-bulky disease who are unable to receive chemotherapy, though relapse rates are higher than with CMT, and CMT is preferred. The role of RT omission in PET-negative patients has been evaluated in the RAPID trial (supporting omission in PET-negative non-bulky favorable disease) and the EORTC/LYSA H10 trial (finding higher recurrence with omission even in PET-negative patients); current guidelines retain CMT as standard while regarding PET-guided RT omission as investigational, particularly in bulky or unfavorable presentations. In the era of highly active novel-agent regimens (Nivo-AVD, BV-AVD), emerging data from NIVAHL and BREACH demonstrate high PET-negativity rates after two cycles, raising the prospect of response-adapted RT reduction, though longer follow-up and randomized evidence are required before omission becomes routine [28,29].

## 13. Frontline Treatment Considerations in Elderly Patients

Elderly and unfit patients with cHL represent a distinct population with unique treatment challenges. Older patients experience inferior outcomes compared with younger patients, partly due to reduced treatment tolerance, higher comorbidity burden, and greater susceptibility to treatment-related toxicity, particularly bleomycin pulmonary toxicity, which is especially concerning in older individuals and those receiving G-CSF support. Standard ABVD is often poorly tolerated in elderly patients, and escalated BEACOPP is generally considered inappropriate due to excessive toxicity.

Several phase II studies have evaluated BV-based approaches adapted for elderly or unfit patients. Forero-Torres et al. evaluated frontline BV monotherapy in patients aged 60 years or older, demonstrating an overall response rate of 92% with manageable toxicity, though grade 3 neuropathy occurred in 30% of patients [30]. Friedberg et al. demonstrated the activity of BV combined with dacarbazine or bendamustine in patients ≥ 60 years ineligible for or declining frontline chemotherapy [31]. Evens et al. evaluated sequential BV followed by AVD in patients ≥ 60 years with stage IIB-IV cHL, demonstrating feasibility, though tolerability varied substantially by comorbidity burden [32]. These regimens capitalize on the high CD30 expression of cHL while avoiding the most problematic toxicities of conventional chemotherapy.

More recently, the elderly subgroup analysis of the phase III SWOG S1826 trial has substantially reshaped the frontline treatment landscape for fit older patients. Among patients aged ≥ 60 years with advanced-stage cHL, nivolumab plus AVD (N-AVD) demonstrated superior progression-free survival and lower non-relapse mortality compared with BV-AVD, while also resulting in significantly lower rates of peripheral neuropathy, febrile neutropenia, sepsis, and infectious complications despite a higher incidence of asymptomatic neutropenia. Treatment discontinuation due to toxicity was also less frequent with N-AVD, supporting its improved overall tolerability. These findings establish N-AVD as the preferred frontline regimen for fit elderly patients who are candidates for anthracycline-based therapy, providing an effective bleomycin-free strategy with a favorable balance between efficacy and safety [33].

The use of PD-1 blockade may offer additional advantages in older patients beyond improved disease control. By eliminating both bleomycin and brentuximab vedotin from the regimen, N-AVD minimizes the risks of pulmonary toxicity and cumulative peripheral neuropathy, two major causes of treatment-related morbidity in the elderly. Furthermore, immune-related adverse events associated with nivolumab are generally infrequent and manageable with appropriate monitoring and corticosteroid therapy, making this approach particularly attractive for older patients with preserved functional status [33].

For very elderly or frail patients who are unable to tolerate combination chemotherapy, emerging evidence suggests that single-agent PD-1 blockade with nivolumab or pembrolizumab may represent a reasonable palliative or chemotherapy-sparing strategy. Although prospective data remain limited and these approaches are not yet considered standard frontline therapy, checkpoint inhibitor monotherapy has demonstrated high response rates, durable disease control, and a favorable toxicity profile in relapsed cHL and in small studies of frail untreated patients, making it an attractive option when curative-intent multi-agent chemotherapy is not feasible. Ongoing prospective studies are expected to better define the role of immunotherapy-alone approaches in this vulnerable population [34,35].

Overall, the optimal frontline management of elderly patients with cHL should be individualized according to chronological age, functional status, comorbidities, frailty assessment, and patient preferences. Comprehensive geriatric assessment and frailty scoring can aid treatment selection and identify patients who may benefit from standard-intensity therapy, modified regimens, or chemotherapy-free approaches. Enrollment of older patients in prospective clinical trials remains strongly encouraged to strengthen the evidence base for this historically underrepresented population [34,36].

## 14. Special Clinical Situations in Frontline cHL Management

cHL presents in several special populations requiring adapted frontline strategies. Adolescents and young adults (AYAs) represent the predominant patient group; the SWOG S1826 trial enrolled patients aged ≥ 12 years and demonstrated superior PFS with nivolumab-AVD versus BV-AVD across all age subgroups, including adolescents, establishing Nivo-AVD as the preferred frontline regimen in fit AYAs with advanced-stage disease. Fertility preservation counseling (sperm cryopreservation in males; oocyte or embryo cryopreservation in females) should be initiated before chemotherapy in all AYA patients. AVD-based regimens are preferred over alkylator-intensive approaches (eBEACOPP, BrECADD) in this population, given lower gonadotoxic risk [10,37].

In pregnant patients with cHL, ABVD administered in the second or third trimester is the best-supported regimen, with retrospective data demonstrating acceptable fetal outcomes. Brentuximab vedotin and PD-1 inhibitors (nivolumab, pembrolizumab) are contraindicated in pregnancy due to embryo–fetal toxicity and disruption of feto-maternal immune tolerance, respectively, and must be deferred until after delivery. Multidisciplinary management involving hematology, maternal–fetal medicine, and neonatology is essential [38].

In people living with HIV, standard ABVD combined with integrase inhibitor-based antiretroviral therapy achieves outcomes comparable to the HIV-negative population when CD4 counts are adequate (≥200 cells/μL). Protease inhibitor-based regimens should be avoided due to CYP3A4-mediated interactions with ABVD and BV components. PD-1 inhibitors in HIV-associated cHL should be used within clinical trials or with close infectious disease co-management, given risks of immune reconstitution inflammatory syndrome and HIV viral rebound [39].

Hodgkin lymphoma arising as post-transplant lymphoproliferative disorder (Hodgkin-type PTLD) is rare, predominantly EBV-driven, and managed initially with reduction in immunosuppression followed by ABVD-based chemotherapy in most cases. PD-1 inhibitors are generally contraindicated in solid organ transplant recipients due to the high risk of irreversible allograft rejection mediated by checkpoint blockade [40].

Patients with cHL and a concurrent or prior malignancy require individualized treatment planning to avoid cumulative organ toxicities and drug–drug interactions. When a concurrent cancer is being treated with checkpoint inhibitors, combining or overlapping with Nivo-AVD carries additive immune-related adverse event risk; BV-AVD or ABVD may represent safer frontline alternatives in this context, guided by multidisciplinary tumor board input.

During the COVID-19 pandemic, consensus recommendations supported omission of bleomycin (substituting AVD) to reduce pulmonary immunosuppression risk, minimization of in-person visits, and avoidance of elective treatment delay in advanced-stage or symptomatic disease. COVID-19 vaccination is recommended for all patients with cHL, ideally before initiating immunosuppressive therapy, with the awareness that immune responses may be attenuated during active treatment [41].

## 15. Toxicity and Survivorship Considerations

As cure rates improve in classical Hodgkin lymphoma (cHL), survivorship considerations have become increasingly important, particularly because many patients are adolescents and young adults expected to live for decades after treatment completion. Consequently, modern frontline treatment strategies aim not only to maximize cure rates but also to minimize long-term treatment-related morbidity and preserve quality of life [1].

Historically, conventional chemotherapy and radiotherapy were associated with substantial late toxicities, including pulmonary injury, infertility, secondary malignancies, cardiovascular disease, and endocrine dysfunction. Bleomycin-induced pulmonary toxicity remains one of the most clinically significant acute and chronic toxicities associated with ABVD, particularly in older patients and those receiving granulocyte colony-stimulating factor support [13]. The omission of bleomycin in BV-AVD and PD-1-based regimens, therefore, represents an important advancement in reducing pulmonary complications.

However, BV-containing regimens introduce different toxicity considerations, most notably peripheral neuropathy. In ECHELON-1, peripheral neuropathy occurred significantly more frequently with BV-AVD compared with ABVD, although many cases improved or resolved with time and dose modification [5,9]. The cumulative neurotoxicity associated with repeated BV exposure is particularly relevant when BV is incorporated into both frontline and salvage settings.

Immune checkpoint inhibitors are associated with a distinct spectrum of immune-related adverse events (irAEs). In the SWOG S1826 trial, nivolumab-AVD demonstrated favorable overall tolerability compared with BV-AVD, with lower rates of febrile neutropenia and neuropathy (Herrera et al., 2024) [10]. Nevertheless, PD-1 blockade may cause immune-mediated toxicities, including thyroid dysfunction, hepatitis, pneumonitis, colitis, adrenal insufficiency, hypophysitis, and dermatologic reactions. Although most irAEs are manageable with corticosteroids and treatment interruption, long-term endocrine dysfunction may persist permanently in some patients.

Fertility preservation remains another major survivorship consideration, particularly in younger patients. Escalated BEACOPP has historically been associated with substantial gonadal toxicity and infertility risk due to high cumulative alkylator exposure [4]. Novel-agent-based approaches such as BV-AVD, nivolumab-AVD, and BrECADD may potentially reduce infertility risk through chemotherapy de-escalation and lower cumulative exposure to gonadotoxic agents, although long-term fertility data remain limited.

Cardiovascular toxicity also remains an important issue due to anthracycline exposure and historical mediastinal radiotherapy. Long-term survivors of cHL have increased risks of coronary artery disease, valvular heart disease, heart failure, and stroke decades after treatment [1]. Emerging strategies aimed at reducing cumulative anthracycline exposure, such as PET-adapted therapy and BrECADD-based approaches, may help reduce long-term cardiotoxicity [18].

Across pivotal frontline trials, from a toxicity standpoint, the currently available frontline regimens for advanced classical Hodgkin lymphoma differ substantially in their adverse event profiles. Nivolumab-AVD (N-AVD) has emerged as one of the best-tolerated intensive regimens, with the SWOG S1826 trial demonstrating grade ≥ 3 neutropenia in approximately 45% of patients but a remarkably low incidence of febrile neutropenia (3–6%), sepsis (~1%), and grade ≥ 2 peripheral neuropathy (~7%), while immune-related adverse events were generally infrequent and manageable, with hypothyroidism occurring in approximately 5% of patients. In contrast, BV-AVD, as reported in ECHELON-1, was associated with substantially higher rates of peripheral neuropathy (67%), including grade ≥ 3 neuropathy (~10%), as well as increased grade ≥ 3 neutropenia (58%) and febrile neutropenia (19%), although it significantly reduced bleomycin-associated pulmonary toxicity compared with ABVD. ABVD remains associated with a relatively lower risk of neuropathy and myelosuppression but is limited by bleomycin-induced pulmonary toxicity, with clinically significant pulmonary events occurring in approximately 3–7% of patients and posing a particular concern in older adults. BrECADD, developed to improve the safety of escalated BEACOPP while maintaining efficacy, substantially decreases organ toxicity compared with its predecessor but remains the most myelosuppressive of the modern regimens, with treatment-related morbidity reported in 42% of patients, grade 4 hematologic toxicity or severe infection in approximately 31%, grade 3–4 organ toxicity in 19%, serious adverse reactions in 39%, and serious febrile neutropenia in 19%. Major toxicities are summarized in Table 3. Overall, these data suggest that N-AVD offers the most favorable balance of efficacy and tolerability, largely by minimizing neuropathy, infectious complications, and pulmonary toxicity, whereas BV-AVD is primarily limited by neurotoxicity, ABVD by bleomycin-related lung injury, and BrECADD by significant hematologic toxicity despite its improved safety compared with escalated BEACOPP [9,10,18].

Secondary malignancies remain a significant concern among long-term survivors, especially following combined-modality therapy involving radiotherapy and alkylator-intensive chemotherapy [14,42]. Breast cancer, lung cancer, thyroid cancer, and therapy-related myeloid neoplasms are among the most recognized late complications. Efforts to reduce radiation exposure in limited-stage disease and minimize chemotherapy intensity represent major priorities in contemporary treatment design [43].

Another increasingly important aspect of survivorship is patient-reported quality of life. Fatigue, neuropathy, cognitive dysfunction, sexual health concerns, psychosocial stress, and financial toxicity may persist long after treatment completion. Incorporation of survivorship-focused care pathways, rehabilitation, fertility counseling, vaccination strategies, cardiovascular screening, and psychosocial support is becoming an essential component of comprehensive cHL management.

Patient-reported outcomes (PROs) and quality-of-life data from pivotal trials provide important complementary evidence to objective toxicity assessments. In the SWOG S1826 trial, PRO analyses demonstrated that patients receiving nivolumab-AVD reported better quality of life scores and lower neuropathy burden compared with BV-AVD, consistent with the clinical toxicity findings [44]. These PRO data reinforce the significance of the toxicity differences between the two regimens and support the preference for nivolumab-AVD in patients where quality of life preservation is a priority. PRO data from ECHELON-1 similarly demonstrated that peripheral neuropathy with BV-AVD had meaningful impacts on patient-reported functional status and quality of life [7,9].

Ultimately, future frontline treatment strategies in cHL will likely focus on achieving the optimal balance between maximizing cure rates and minimizing long-term toxicity. Biomarker-driven therapy selection, PET-adapted treatment, chemotherapy reduction strategies, and incorporation of highly effective immunotherapy-based approaches may collectively improve survivorship outcomes while preserving the excellent disease control achieved in modern cHL therapy.

## 16. Relapse Following PD-1 Inhibitor-Based Frontline Therapy

As nivolumab-AVD becomes increasingly adopted as the frontline standard, the clinical question of optimal salvage strategies for patients who relapse following PD-1 inhibitor-based frontline therapy is of growing practical importance. The optimal management of PD-1 inhibitor-pretreated relapsed/refractory cHL presents unique challenges, as PD-1 blockade in the frontline setting may alter the tumor immune microenvironment and potentially affect the efficacy of subsequent PD-1-based salvage approaches [45].

For transplant-eligible patients relapsing after frontline chemoimmunotherapy, the goal of salvage therapy is to achieve complete metabolic response and proceed to high-dose chemotherapy followed by autologous stem cell transplantation (ASCT). Established platinum-based salvage regimens include ICE (ifosfamide, carboplatin, etoposide), DHAP, ESHAP, and GDP, achieving ORR of 60–75%. The NICE regimen (nivolumab + ICE) demonstrated ORR 100% and CR 88% in high-risk relapsed/refractory cHL, providing a highly effective bridge to ASCT. For patients who relapsed after frontline Nivo-AVD, BV-based salvage (BV monotherapy or BV + bendamustine) is preferred at first relapse over PD-1 inhibitor re-challenge, given the risk of acquired checkpoint resistance. Post-ASCT consolidation with BV (AETHERA trial) significantly improved PFS in high-risk patients and should be considered in eligible cases [46,47,48].

Allogeneic HSCT (alloHSCT) is reserved for patients who relapse after or are ineligible for ASCT, exploiting a graft-versus-Hodgkin lymphoma effect via donor T-cells. Reduced-intensity conditioning is preferred to minimize non-relapse mortality while preserving graft-versus-lymphoma activity, with long-term PFS of approximately 25–35% in multiply relapsed/refractory cHL. In patients previously treated with PD-1 inhibitors, an interval of at least 2–4 weeks between the last checkpoint inhibitor dose and alloHSCT conditioning is recommended, as shorter intervals have been associated with hyperacute graft-versus-host disease and life-threatening immune-mediated complications. AlloHSCT remains the only established curative option in the post-ASCT relapse setting; all eligible patients should be evaluated at experienced centers and enrolment in prospective studies is encouraged [49,50].

A critical question is whether re-challenge with PD-1 inhibitors is appropriate in patients who relapse after checkpoint inhibitor-containing frontline therapy. Patients who achieve durable responses and relapse after a prolonged interval may still benefit from checkpoint inhibitor re-challenge, potentially in combination with other agents. In contrast, early relapse during or shortly after PD-1 inhibitor-based frontline therapy may reflect primary resistance to checkpoint inhibition, and alternative salvage strategies including BV-based regimens should be prioritized [45]. Prospective studies specifically evaluating salvage strategies in the post-nivolumab-AVD setting are urgently needed to define optimal treatment algorithms for this evolving patient population.

## 17. Future Directions

The therapeutic landscape of classical Hodgkin lymphoma (cHL) continues to evolve rapidly, with ongoing research increasingly focused on improving precision medicine approaches while minimizing long-term treatment-related toxicity. Biomarker-driven therapy selection using molecular and immunologic profiling is a high-priority research area. 9p24.1 amplification, PD-L1 expression, EBV status, and tumor microenvironment composition are among the biomarkers under prospective evaluation for their ability to guide frontline treatment selection [6,11,51].

Circulating tumor DNA (ctDNA) monitoring is gaining significant interest as a tool for early-response assessment, minimal residual disease (MRD) detection, and prediction of relapse before radiographic progression becomes clinically evident. Prospective studies, including Spina et al., demonstrated that the ctDNA level at diagnosis correlates with stage and IPS, while a reduction in ctDNA plasma levels after initial treatment is highly correlated with prognosis in cHL [52]. Targeted genotyping of ctDNA has been validated as a prospective monitoring tool in cHL, with persistent ctDNA signaling identifying patients at higher relapse risk [53]. Integration of ctDNA into treatment algorithms may enable more precise, biologically informed PET-adapted strategies in future trials.

Notably, ctDNA analyses from SWOG S1826 demonstrated that molecular tumor burden assessment was superior to PET response in predicting outcomes with Nivo-AVD and BV-AVD, suggesting ctDNA may complement or eventually refine PET-adapted strategies in future frontline trials [22].

Chemotherapy minimization strategies are being explored through incorporation of highly active immunotherapy-based regimens. Ongoing studies are evaluating whether chemotherapy can be further reduced or even eliminated in selected patients with biological features predicting exceptional sensitivity to checkpoint inhibition or targeted therapy [25,26].

Beyond PD-1 blockade, novel immunotherapeutic approaches, including bispecific antibodies and chimeric antigen receptor T-cell (CAR-T) therapy, are under active investigation in earlier lines of therapy. The bispecific antibody AFM13, which targets CD30 on Reed–Sternberg cells and CD16A on natural killer cells, has demonstrated single-agent activity in relapsed/refractory cHL and remarkable response rates (ORR 89%) when combined with cord blood-derived NK cells, supporting further evaluation in earlier disease settings [54,55]. CD30-directed CAR-T-cell therapy (CD30.CAR-T) has demonstrated favorable safety and promising antitumor activity (ORR 72%, CR 59%) in relapsed/refractory cHL in phase I/II studies, and is being evaluated in combination with nivolumab after frontline therapy failure in the ACTION trial [56,57]. These emerging modalities may eventually expand treatment options across different stages of cHL.

Additionally, multiple studies are evaluating radiotherapy omission strategies in selected PET-negative patients in an effort to decrease long-term cardiovascular toxicity and secondary malignancies. Beyond PD-1 blockade, novel immunotherapeutic approaches, including bispecific antibodies and cellular therapies, are also under active investigation and may eventually expand treatment options across different stages of disease. Collectively, these advances reflect a broader shift toward individualized treatment strategies in cHL that aim to maximize cure while optimizing long-term survivorship and quality of life.

## 18. Conclusions

Frontline treatment of classical Hodgkin lymphoma has undergone a major transformation with the integration of brentuximab vedotin and PD-1 inhibitors into first-line therapy. BV-AVD established the first survival advantage over ABVD in advanced-stage disease, while nivolumab-AVD demonstrated superior progression-free survival with improved tolerability compared with BV-AVD. PET-adapted therapy remains central to treatment optimization, while novel-agent incorporation continues to evolve in both advanced-stage and limited-stage disease.

Future therapeutic strategies will likely focus on chemotherapy reduction, biomarker-driven approaches, and survivorship optimization while preserving the exceptional cure.

## Figures and Tables

**Table 1:** Frontline trials incorporating novel agents in advanced-stage.

Trial	Population	Regimen/Comparator	Phase/Status	Median Follow-Up	Key Results/Outcomes
SWOG S1826	Stage III–IV cHL, age ≥ 12 years	Nivolumab + AVD vs. BV + AVD X 6 cycles	Phase III; practice-changing	Median 37.2 months	N-AVD significantly improved PFS versus BV-AVD; HR 0.42. Lower treatment discontinuation and fewer serious toxicities. Established N-AVD as a new frontline standard for advanced cHL.
GHSG HD21	Advanced-stage cHL	BrECADD vs. escalated BEACOPP	Phase III	Median 48.9 months	BrECADD improved tolerability while maintaining or improving disease control versus eBEACOPP. 4-year PFS reported around 94% in updated analyses.
ECHELON-1	Previously untreated stage III–IV cHL	BV-AVD vs. ABVD	Phase III	Median 82.1 months	First frontline novel-agent trial showing durable benefit over ABVD. 6-year OS: 93.9% vs. 89.4%; HR 0.59. Sustained PFS advantage with reduced relapse risk.
SGN35-027 (AN + AD cohort)	Untreated advanced-stage cHL	BV + nivolumab + doxorubicin + dacarbazine (vinblastine omitted)	Phase II	Median 27.8 months	ORR approximately 93–95%, CR approximately 88–89%; 2-year PFS around 88%. Demonstrated feasibility of a vinblastine-free regimen.
Older-patient BV studies	Older/unfit untreated advanced cHL	Sequential BV → AVD or BV + dacarbazine	Phase II	Variable (18–36 months)	High response rates with improved tolerability for elderly patients; not considered standard for fit younger patients.

Abbreviations: cHL = classical Hodgkin lymphoma; N-AVD = nivolumab, doxorubicin, vinblastine, dacarbazine; BV-AVD = brentuximab vedotin, doxorubicin, vinblastine, dacarbazine; BrECADD = brentuximab vedotin, etoposide, cyclophosphamide, doxorubicin, dacarbazine, dexamethasone; eBEACOPP = escalated bleomycin, etoposide, doxorubicin, cyclophosphamide, vincristine, procarbazine, prednisone; ABVD = doxorubicin, bleomycin, vinblastine, dacarbazine; PFS = progression-free survival; OS = overall survival; ORR = overall response rate; CR = complete response; HR = hazard ratio; AVD = doxorubicin, vinblastine, dacarbazine; BV = brentuximab vedotin.

**Table 2:** Frontline trials incorporating novel agents in limited-stage cHL.

Trial	Population	Regimen/Comparator	Phase/Status	Median Follow-Up	Key Results/Outcomes
NIVAHL	Early-stage unfavorable cHL	Sequential or concomitant nivolumab + AVD followed by radiotherapy	Randomized Phase II	Median 41 months	CR rates approximately 90–94%; 12-month PFS 98–100%. Long-term follow-up demonstrated excellent durability with minimal progression events.
BREACH	Early-stage unfavorable cHL	BV-AVD vs. ABVD followed by involved-node RT	Randomized Phase II	Median 28.0 months	PET-negative rate after 2 cycles significantly higher with BV-AVD (~82%). Demonstrated improved early metabolic response and excellent 2-year PFS.
RADAR	Early-stage favorable cHL	PET-adapted BV-based strategy with omission of radiotherapy in selected patients	Phase II	Still recruiting	Early data demonstrated high PET-negativity rates and feasibility of response-adapted radiotherapy reduction strategies with encouraging progression-free survival outcomes.
SGN35-027 (early-stage cohort)	Early-stage cHL	BV + nivolumab + doxorubicin + dacarbazine	Phase II	27.9	ORR approximately 95%, CR approximately 92%. Suggested highly active chemotherapy de-escalated approach.
Pilot BV-AVD limited-stage studies	Favorable/unfavorable early-stage cHL	BV-AVD ± radiotherapy	Early phase studies	Variable (12–24 months)	Demonstrated high PET-negativity and encouraging short-term PFS, supporting ongoing de-escalation strategies.

Abbreviations: cHL = classical Hodgkin lymphoma; BV-AVD = brentuximab vedotin, doxorubicin, vinblastine, dacarbazine; ABVD = doxorubicin, bleomycin, vinblastine, dacarbazine; BV = brentuximab vedotin; AVD = doxorubicin, vinblastine, dacarbazine; RT = radiotherapy; PET = positron emission tomography; CR = complete response; ORR = overall response rate; PFS = progression-free survival.

**Table 3:** Comparative grade ≥ 3 adverse event profiles of main frontline regimens in advanced-stage cHL.

Adverse Event	N-AVD/(SWOG S1826)	BV-AVD/(ECHELON-1)	ABVD/(ECHELON-1)	BrECADD/(HD21)
Febrile neutropenia (Gr ≥ 3)	3%	19%	19%	13%
Peripheral neuropathy (Gr ≥ 3)	3%	10%	3%	7%
Neutropenia (Gr ≥ 3)	~45%	~58%	~54%	~60%
Pulmonary toxicity/bleomycin-related (Gr ≥ 3)	Not applicable (bleomycin-free)	Not applicable (bleomycin-free)	3–7%	Not applicable
Immune-related AEs (Gr ≥ 3)—irAE	~13%	Not applicable	Not applicable	Not applicable
Hypothyroidism (all grades)	~10%	<1%	<1%	<1%
Pneumonitis (Gr ≥ 3)	~2%	<1% (non-bleomycin)	1–3% (bleomycin)	<1%
Infection (Gr ≥ 3)	~8%	~18%	~15%	~19%
Treatment discontinuation (toxicity)	~7%	~13%	~6%	~10%
Infertility/gonadotoxicity risk	Low	Low	Low	Lower vs. eBEACOPP
Secondary malignancy risk (long-term)	Under evaluation	Comparable to ABVD	Low	Lower vs. eBEACOPP

Abbreviations: N-AVD = nivolumab, doxorubicin, vinblastine, dacarbazine; BV-AVD = brentuximab vedotin, doxorubicin, vinblastine, dacarbazine; ABVD = doxorubicin, bleomycin, vinblastine, dacarbazine; BrECADD = brentuximab vedotin, etoposide, cyclophosphamide, doxorubicin, dacarbazine, dexamethasone; AE = adverse event; Gr = grade; irAE = immune-related adverse event; eBEACOPP = escalated bleomycin, etoposide, doxorubicin, cyclophosphamide, vincristine, procarbazine, prednisone.

## Data Availability

No new data were created or analyzed in this study.
